# Predictive Model of Quality of Life in Patients with Parkinson’s Disease

**DOI:** 10.3390/ijerph19020672

**Published:** 2022-01-07

**Authors:** Eduardo Candel-Parra, María Pilar Córcoles-Jiménez, Victoria Delicado-Useros, Marta Carolina Ruiz-Grao, Antonio Hernández-Martínez, Milagros Molina-Alarcón

**Affiliations:** 1 Department of Nursing, Physiotherapy and Occupational Therapy, Faculty of Nursing, University of Castilla-La Mancha, Av. de España, s/n, 02001 Albacete, Spain; Eduardo.Candel@uclm.es (E.C.-P.); pilar.corcoles@uclm.es (M.P.C.-J.); victoria.delicado@uclm.es (V.D.-U.); Marta.Ruiz@uclm.es (M.C.R.-G.); 2 Instituto de Investigación en Discapacidades Neurológicas (IDINE), University of Castilla-La Mancha, Av. de España, s/n, 02001 Albacete, Spain; 3 Department of Nursing, Physiotherapy and Occupational Therapy, Faculty of Nursing, University of Castilla-La Mancha, 13071 Ciudad Real, Spain

**Keywords:** Parkinson’s disease, quality of life, PDQ-39, model predictive

## Abstract

Parkinson’s disease is a chronic, progressive, and disabling neurodegenerative disease which evolves until the end of life and triggers different mood and organic alterations that influence health-related quality of life. The objective of our study was to identify the factors that negatively impact the quality of life of patients with Parkinson’s disease and construct a predictive model of health-related quality of life in these patients. Methods: An analytical, prospective observational study was carried out, including Parkinson’s patients at different stages in the Albacete Health Area. The sample consisted of 155 patients (T0) who were followed up at one (T1) and two years (T2). The instruments used were a purpose-designed data collection questionnaire and the “Parkinson’s Disease Questionnaire” (PDQ-39), with a global index where a higher score indicates a worse quality of life. A multivariate analysis was performed by multiple linear regression at T0. Next, the model’s predictive capacity was evaluated at T1 and T2 using the area under the ROC curve (AUROC). Results: Predictive factors were: sex, living in a residence, using a cane, using a wheelchair, having a Parkinson’s stage of HY > 2, having Alzheimer’s disease or a major neurocognitive disorder, having more than five non-motor symptoms, polypharmacy, and disability greater than 66%. This model showed good predictive capacity at one year and two years of follow-up, with an AUROC of 0.89 (95% CI: 0.83–0.94) and 0.83 (95% CI: 0.76–0.89), respectively. Conclusions: A predictive model constructed with nine variables showed a good discriminative capacity to predict the quality of life of patients with Parkinson’s disease at one and two years of follow-up.

## 1. Introduction

Parkinson disease (PD) is a progressive, chronic, and disabling neurodegenerative disease with important mood disturbances and resulting changes in lifestyle. Hospital clinical records indicate a worldwide prevalence of between 100 and 300 cases per 100,000 inhabitants [1,2]. In terms of pathology, PD is defined by the progressive loss of dopaminergic neurons in the substantia nigra and the presence of inclusions called Lewy bodies. No biological markers exist for its diagnosis; therefore, a diagnosis of PD is clinical and established from the clinical history and examination of the patient. However, there are several markers associated with cognitive impairment in PD, including clinical, neuropathological, genetic, and neuroimaging markers [3]. Cognitive impairment is an important aspect of PD to consider as it has a severe negative effect on health and health-related quality of life (HRQoL).

From a clinical point of view, two large groups of disorders are distinguished in PD. The first group refers to motor symptoms (MS), such as tremors, poor or slow movements (akinesia), increased muscle tone or rigidity, and abnormal involuntary movements (dyskinesias). The combination of tremor, rigidity, akinesia-bradykinesia (absent-slow movements, especially of complex voluntary movements), and impairment of postural reflexes is referred to as “parkinsonian syndrome”. In particular, slow movements and rigidity limit normal function, and this stiffness is responsible for muscle pain in these patients. However, the most serious motor problem is akinesia, with varying degrees of movement and changes in position [3,4]. The second group of disorders refers to non-motor symptoms (NMS), including autonomic dysfunction (constipation, hyperhidrosis), sensory dysfunction (paresthesia, pain), and psychological disorders (depression, major neurocognitive disorder) [4]. Mood and cognitive disorders affect 80.6% of patients with PD and have a severe negative effect on perceived HRQoL [4,5,6] and their caregivers [7]. NMS are common in late-stage PD and generally have a greater impact on HRQoL in PD than MS. NMS associated with a decline in HRQoL include disrupted sleep architecture, constipation, hyposmia, anxiety, depression, fatigue, chronic pain, impaired speech, and difficulty swallowing [3,7]. Depression is the neuropsychiatric disorder that has shown the greatest negative influence on HRQoL in these patients [5,6,8]. Moreover, these symptoms are directly associated with HRQoL, regardless of the severity of the MS. The variables considered prognostic are depressive symptoms, insomnia, and a low degree of independence in relation to the severity of the disease. Furthermore, around half of patients with PD report a lack of energy related to depressive symptoms [9,10].

The Hoehn and Yahr (HY) stages are used to classify the evolution and progression of PD and are based on changes in motor symptoms, identifying five stages from least to greatest involvement [11].

At this time, there is no curative treatment for PD, so therapies focus on improving symptoms, delaying motor complications, and prolonging patients’ autonomy for as long as possible. Pharmacological, surgical, or rehabilitative treatment can be used, and all these can be combined. Levodopa and other dopamine agonist drugs are used among the pharmacological treatments [12].

Chronic diseases affect all aspects of an individual’s life and generally encompass various elements that influence wellbeing and satisfaction with life. Hence, HRQoL is understood as the perception and evaluation by the patients themselves of the impact on their lives caused by their disease and its consequences. This includes physical, mental, and social aspects related to the state of health and care, in addition to the global perceptions about health and other personal constructs [13,14].

PD has a considerable impact on HRQoL, which has been measured using a variety of instruments. The instrument most used in the different studies is the PDQ-39 [15]. This questionnaire was developed exclusively for patients with Parkinson’s disease (PD) and it evaluates 39 parameters in eight groups of issues: mobility, activities of daily living, emotional wellbeing, stigma of the disease (stigma), back in the next, cognitive, communication, and bodily discomfort. The number of evaluated parameters in each group is from 3 to 10. The respondent has to choose one of five possible answers [3]. PD influences all aspects of a person’s life, including wellbeing and satisfaction with life. In the absence of a cure, one of the main objectives of care is to improve or maintain the HRQoL of a patient with PD as it is negatively affected in these patients by multiple factors and is related to various clinical variables, as well as illness duration. A longer duration of PD supposes a lower HRQoL. PD results in a series of changes due to stress and related to MS (difficulty in movement and slowness, among others), as well as to NMS (constipation, dysphagia, fatigue, pain, sleep disorders, and sexual dysfunction, among others), mood disorders (depression and anxiety), disability (difficulty in personal hygiene, falls, communication), and social dysfunction. Each of these factors, and the presence of several simultaneously, has a cumulative effect on decreasing HRQoL [16].

Evaluating the HRQoL in patients with PD is essential, considering the multiple variables that affect it, along with the complications and adverse effects of treatment, all of which increase the difficulty of assessing the clinical picture. The PDQ-39 questionnaire examines HRQoL, taking into account the subjective opinion of the individual patient and the different factors that may affect them, including physical, functional, psychological, and social factors [15,16]. Currently, few studies have constructed predictive models of HRQoL in patients with PD; most factors that predict the evolution of HRQoL include baseline disease and sociodemographic characteristics, and these factors are difficult to modify [17]. The improvement of depression and postural instability influence the quality of life of patients with PD; therefore, focusing on controlling some of the influencing variables should be the most important objective in improving HRQoL [3]. Depressive symptoms have been identified as the most important determinants in all predictive models. Nonetheless, a complete understanding of HRQoL, its determining factors, and their interrelationships will allow the development of intervention strategies in these patients in order to improve their HRQoL [18,19,20,21]. Furthermore, evaluating the evolution of HRQoL in patients with PD over the years will provide valuable information.

The objective of our study was to identify the factors that have a negative association with the quality of life of patients with Parkinson’s disease and to construct a predictive model of HRQoL in these patients.

## 2. Materials and Methods

### 2.1. Population and Study Design

An analytic, prospective observational study was carried out on patients diagnosed with PD and seen at the Movement Disorder Unit (UTM, abbreviation for the Movement Disorder Unit in Spanish) of the Neurology service for the Albacete Integrated Management Area (GAI, abbreviation for the Albacete Integrated Management Area in Spanish). All patients with PD seen at the UTM and who agreed to participate in this study were included. Patients who did not have an adequate comprehension level of Spanish were excluded.

The estimated sample size was calculated based on an estimated PD prevalence in the general population of 187 cases per 100,000 inhabitants [22] and the 2015 population of the Albacete Health Area (414,892 inhabitants), giving an estimated 776 persons diagnosed with PD. If the mean score and standard deviation of HRQoL were similar to that obtained in the study by Rahman (mean = 32.4; SD = 16.3) [23], then for a 95% confidence level and a precision of ±2.5, the estimated sample size was 135 subjects (EpiDat v3.1. Epidemiology Service of the Dirección Xeral de Saúde Pública, Santiago de Compostela, Spain).

This sample size was increased by 15% to account for possible non-responders, giving a final sample size of 155 subjects. From January 2015 to December 2016, participants were selected consecutively until the estimated sample size was met. From the moment of inclusion, subjects were followed for two years with repeated HRQoL measurements: at baseline (T0), one year (T1), and at two years (T2).

With regard to the inclusion criteria, all patients diagnosed with Parkinson’s disease were included in the movement disorder unit (UTM) consultation until the sample was completed, and they gave their consent to participate once they were admitted and we had explained the purpose of this study. Patients diagnosed with Parkinson’s with cognitive impairment reflected in the medical history and who could not be contacted, either directly or via their main caregiver, by mail or telephone, were excluded.

### 2.2. Study Variables

The sociodemographic and disease-related variables recorded were: age, sex, marital status, living situation, employment status, education level. The clinical variables used were the categorized HY stages (3–4 vs. 1–2), duration of PD (measured from the date of diagnosis to the date of inclusion in the study), deep brain stimulation (DBS; yes vs. no), polypharmacy (categorized as consumption of up to 4 vs. >4 drugs), major neurocognitive disorder, number of motor symptoms (categorized as >3 symptoms vs. ≤3 symptoms), and number of non-motor symptoms (categorized as >5 symptoms vs. ≤5 symptoms). In addition, socio-sanitary variables were collected such as whether they had a caregiver, degree of disability (according to the scales approved by Royal Decree 1971/1999 of December 23, Procedure for the recognition, declaration, and qualification of the degree of disability https://www.boe.es/eli/es/rd/1999/12/23/1971/con) (accessed on 26 November 2021), or benefits from the care system for dependency, mortality, and disability (categorized as >66% vs. ≤66%). The dependent variable was HRQoL.

### 2.3. Measuring Instruments

Data collection questionnaire: Purpose-designed.

The “Parkinson’s Disease Questionnaire” (PDQ-39) [15] was used to measure HRQoL and contains 39 items covering 8 dimensions. The dimensions are mobility, activities of daily living (ADL), emotional wellbeing, social support, stigma, communication, cognitive state, and pain. Each item has five options (from 0 = never to 4 = always or unable to do so). The results were calculated as a percentage—the scores of the items for each dimension were added, multiplied by 100, and divided by the maximum dimension score. A higher score indicates a worse HRQoL. A global index can be obtained by calculating the mean of the scores for each dimension (PDQ summary index, PDQ-39 SI), which summarizes the result of the scale. This questionnaire has been used previously in clinical trials in which the variations in the different dimensions have been congruent with the clinical evaluations made using the usual scales for PD, indicating an adequate sensitivity to change in the clinical features of the patients. The requirements of this questionnaire included the need to complete it through a personal interview the first time at recruitment or baseline [15].

### 2.4. Study Procedures

First, a pilot of the data collection phase was carried out with ten patients to assess the validity of the purpose-made questionnaire for data collection in the present study. An information sheet was prepared on the study’s objectives, and written consent was re-quested for patients who wished to participate in the study. Then, data collection was carried out at the UTM once voluntary informed consent was obtained, interviewing the patients or main caregivers and consulting the medical records. The questionnaires were repeated at one and two years by telephone interview or by sending it by post.

### 2.5. Statistical Analysis

**Figure 1:**
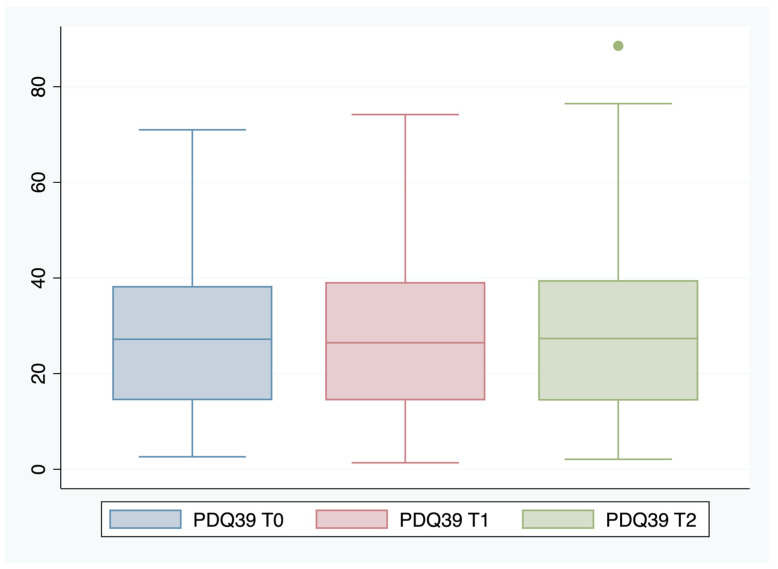
PDQ-39 scores at baseline (T0), 12 months (T1), and 24 months (T3).

Data were analyzed using IBM SPSS v.24 (IBM, Armonk, NY, USA) and STATA15 (StataCorp., College Station, TX, USA). A descriptive statistical analysis of each variable was performed, calculating absolute and relative frequencies for the qualitative variables, and mean and standard deviation (SD) for the quantitative variables. The Kolmogorov–Smirnov and Levene tests were used to check the fit of the empirical data to a normal distribution and the homoscedasticity of the distributions. Next, the 95% confidence intervals (CI) were calculated. To determine the relationship between the different factors and HRQoL, the mean difference (MD) of scores was calculated by linear regression. Later, the adjusted mean difference (aMD) was calculated by multiple linear regression in the T0 cohort. The automatic backward and forward stepwise procedure was used for the multivariate analysis. Using Lemeshow’s statistical criteria, associations with *p*-values of <0.25 in the bivariate analysis with HRQoL were chosen for inclusion in the multivariate linear regression model [24,25]. The predictive model was created on the T0 cohort, and, later, its predictive capacity on T1 and T2 was evaluated. We used values above the 75th percentile of the PDQ-39 value distribution as a cut-off point, around 40 points at the three time points (T0, T1, and T2) (Figure 1). Finally, the areas under the ROC curve (AUROC) for the T0, T1, and T2 cohorts were estimated for the predictive model constructed. In order to assess the prediction qualitatively, we used Swets’s criteria: range 0.5–0.6 (bad), 0.6–0.7 (poor), 0.7–0.8 (satisfactory), 0.8–0.9 (good), and 0.9–1.0 (excellent) [26].

### 2.6. Ethical Aspects

This study was conducted in accordance with the principles of the Declaration of Helsinki regarding studies involving human subjects and in line with Law 14/2007 for biomedical research. Furthermore, the principles of confidentiality and anonymity in the treatment of data and presentation of results were respected following Regulation (EU) 2016/679 of the European Parliament and of the Council, 27 April 2016, on the protection of natural persons with regard to the processing of personal data and on the free movement of such data. This study was approved by the Clinical Research Ethics Committee for the Albacete Health Area (Report 03/11) and the Clinical Research Commission of the GAI of Albacete. The authors declare no conflict of interests.

## 3. Results

A final 155 subjects were included with valid data: at one year (T1), 148 subjects were followed up, and at two years (T2), 141 subjects were followed up. All losses were due to death. All descriptive sociodemographic data refer to baseline characteristics (T0) and are presented in Table 1. Figure 1 shows the scores obtained at the three time points (T0, T1, and T2).

The patients included in this study had mean duration of PD of 9.71 years (SD = 6.46), 95% CI 8.68–10.73. Twenty-nine patients (18.8%) had been diagnosed with PD before the age of 50 years.

The mean MS present among the patients was 2.77 (SD = 1.26, 95% CI 2.57–2.97). The mean number of NMS was 4.47 (SD = 2.24, 95% CI 4.12–4.83), and the mean number of MS and NMS present at the same time was 7.25 (SD = 3.09, 95% CI 6.76–7.74).

Next, a bivariate analysis was carried out using linear regression between the potential factors associated with quality of life in persons with PD in the initial assessment. A statistically significant relationship was observed for all the variables studied (*p* < 0.05) except for sex and the use of surgical intervention for neurostimulation (Table 2).

Next, we performed a multivariate analysis of all the factors with a *p*-value <0.25 for an association with quality of life in the bivariate analysis. After performing a backward and forward stepwise analysis, the predictive model was constructed with nine predictors. The predictive factors were: sex, living in a residence, using a cane, using a wheelchair, having a Parkinson’s stage of HY >2, having Alzheimer’s disease or a major neurocognitive disorder, having more than five NMS, polypharmacy, and a disability greater than 66% (Table 2).

**Figure 2:**
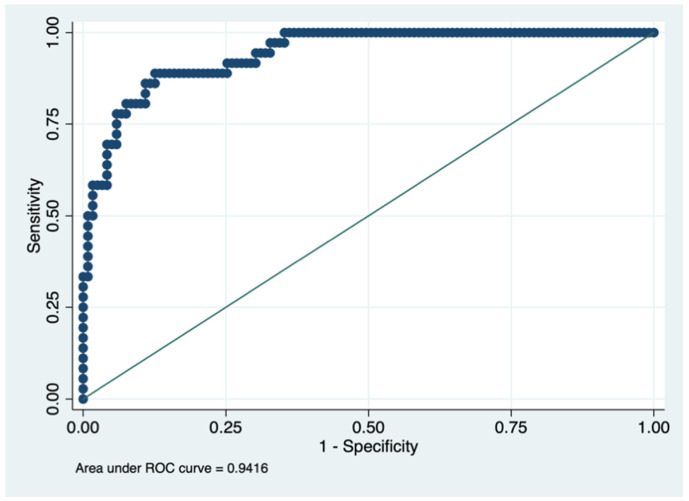
Predictive capacity for the cut-off point >40 points for the PDQ-39 questionnaire (area under the ROC curve (AUROC)). Figure legend. AUROC to determine the predictive ability of the model in the validation cohort, representing the sensitivity on the y-axis and specificity on the x-axis.

The adjusted R-square of this model was 0.622, and the AUROC was 0.94 (95% CI: 0.89–0.97) for a cut-off of >40 points for quality of life using the PDQ-39 (Figure 2).

**Figure 3:**
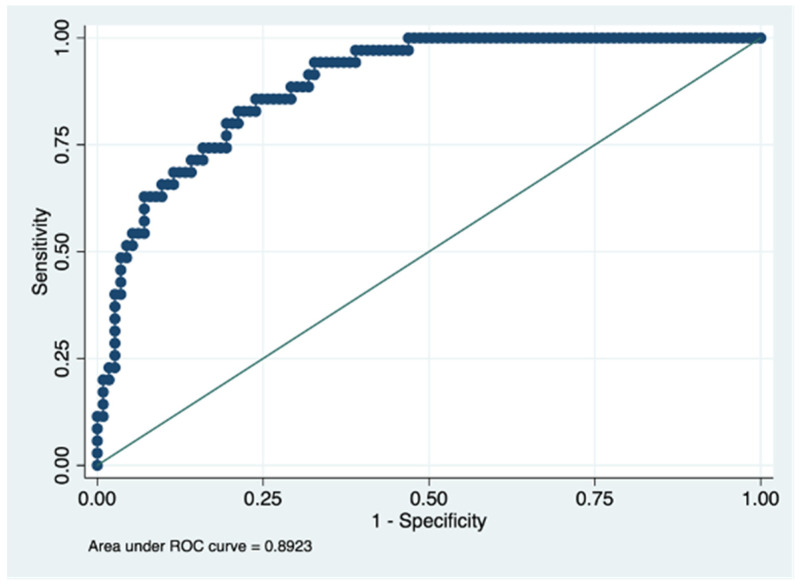
Predictive capacity for the cut-off point >40 points for the PDQ-39 questionnaire at one year. Figure legend. AUROC to determine the predictive ability of the model in the validation cohort, representing the sensitivity on the y-axis and specificity on the x-axis.

**Figure 4:**
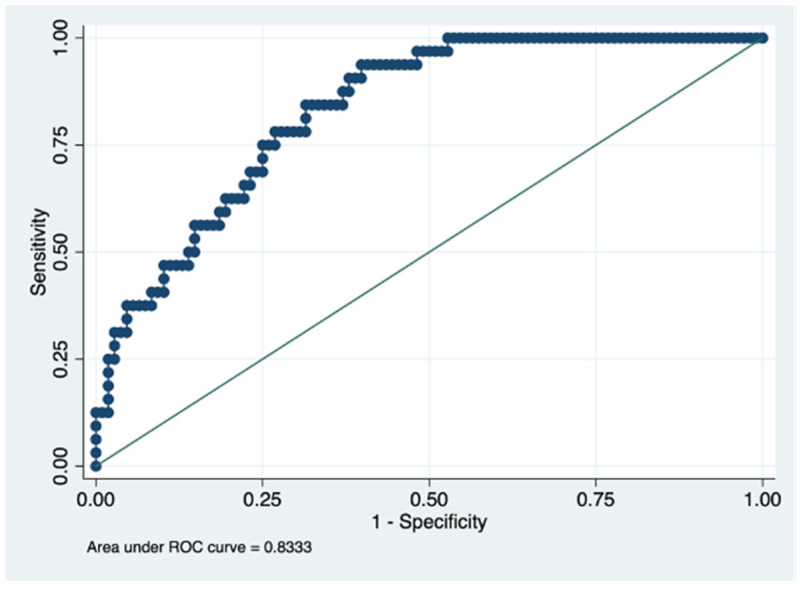
Predictive capacity for the cut-off point >40 points for the PDQ-39 questionnaire at two years. Figure legend. AUROC to determine the predictive ability of the model in the validation cohort, representing the sensitivity on the y-axis and specificity on the x-axis.

Next, the predictive capacity of this model was evaluated at one and two years of follow-up, finding an AUROC of 0.89 (95% CI: 0.83–0.94) and 0.83 (95% CI: 0.76–0.89), respectively (Figure 3 and Figure 4). Both in the initial patient evaluation and the follow-up evaluations at one and two years, the AUROC values were good according to Swet’s qualitative criteria [24].

**Figure 5:**
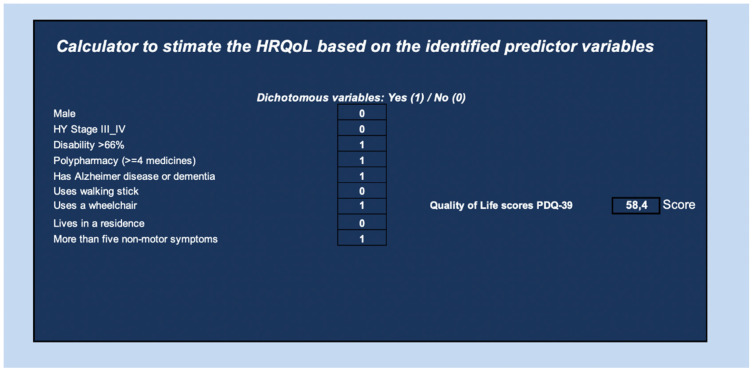
Quality of life scores PDQ-39. Automatic calculator.

Finally, we designed a calculator to estimate the HRQoL score based on the identified predictor variables. Our model predicts the global HRQoL score based on the presence or absence of these factors. For example, a female patient with HY stage III-IV, disability >66%, polypharmacy ≥4 drugs, has a diagnosis of a major neurocognitive disorder, more than five NMS, uses a wheelchair, and does not live in a residence would have an HRQoL score of 58.4 points (Figure 5).

## 4. Discussion

In the present study, we aimed to identify the factors that negatively correlated with the quality of life of patients with Parkinson’s disease and construct a predictive model of HRQoL in these patients. Notably, both psychological and physical activity interventions that patients can perform should be taken into account. Cholewa et al., 2016, advised that remaining active at work and physical therapy helped reduce symptoms and improve patients’ HRQoL [25]. Oguh et al., 2014, also noted the importance of regular physical exercise to improve HRQoL in patients with PD, as well as to lower the caregiver burden and have less cognitive impairment one year later [27]. In addition, Simpson et al., 2014, indicated that physical and mental rehabilitation could be beneficial for the HRQoL of patients with PD if it is well-planned [28]. However, our study found no statistically significant relationship with global HRQoL for patients who underwent physical therapy, occupational therapy, speech therapy, psychological therapy, cycling, or physical-aerobic maintenance activities. This lack of findings was likely due to the small number of PD patients who perform these activities. The activity of choice of the patients in our study was going for a walk, and this activity was related to a better HRQoL: results consistent with those found by Luciana Scalzo et al., 2012 [29]. Likewise, in the study by Duncan et al., 2011, the importance of ensuring mobility was affirmed, especially as it is a determining factor in predictive models of HRQoL [30].

Ueno et al., 2020, concluded that the treatment of MS and, more importantly, NMS could contribute to improving HRQoL in patients with PD [31]. The number of MS has a statistically significant relationship with worse HRQoL, except for tremor. We also observed a significant statistical relationship for NMS, with more NMS associated with worse HRQoL. The studies by Erro et al., 2016, and Prakash et al., 2016, indicated that the total load of NMS predicted a higher score in the global HRQoL, and its influence on HRQoL scores was greater than the influence of MS; sleep and mood had a very significant impact in particular [32,33]. In our study, patients received pharmacological treatment to alleviate the effect of MS and NMS. Moreover, patients with polypharmacy (more than four drugs) were observed to have statistically significant worse HRQoL. Moreover, the study by Martínez-Fernández et al., 2016, observed that drug therapy had adverse effects such as headaches, nausea, dry mouth, constipation, drowsiness, fatigue, dizziness, nightmares, hypotension, and motor fluctuations [34].

Major neurocognitive disorder was also associated with worse overall HRQoL in the results of our study. Similarly, Schiavolin et al., 2017 [8], stated that psychosocial difficulties and cognitive and motor impairment were the most important predictors that influence a lower HRQoL. In contrast, family relationships and social relationships were found to have a very positive influence.

Another important factor is the HY stage in which the patients are found. Strupp et al., 2018, found that patients who have a stage >2 have worse HRQoL and advised physical exercise to avoid impairment of functional mobility [35]: results that are in line with ours. This study shows how HRQoL in these patients showed different scores, depending on the clinical evolution of the disease and different social and individual factors. Determining the factors that help predict the evolution of the patients’ HRQoL assists in formulating preventive or therapeutic interventions for these factors to control or modulate their appearance. In the case of non-modifiable factors, supportive care for patients and their families could be added. Concerning a worse HRQoL in women, these findings indicate that interventions directed specifically to women should be developed. The influence of NMS on global HRQoL should also be highlighted—many NMS are preventable or treatable with nursing interventions within the framework of the multidisciplinary team. Thus, providing a path of action to improve the HRQoL of PD patients with nursing care through action on NMS. Specifically, interventions to prevent or treat constipation, urinary incontinence, or dysphagia, as well as educational interventions and training programs aimed at both patients and caregivers, can be carried out independently by nurses in the community setting or other settings.

In the context of a society with a large number of older people and with a sizable number affected by PD, an effort should be made to develop health policies that affect and improve HRQoL as it is an aspect that generates financial costs and causes suffering for patients and their families.

Among the limitations of the present study, the sociodemographic and social health variables have been obtained by the testimony of the subjects in the sample, so there could be some memory bias or the concealment of sensitive data.

Additionally, it is possible that the recruitment system through face-to-face assistance to consultations in this unit reduced the inclusion in the study of patients in the more advanced stages, since having worse mobility conditions would make it more difficult for them to travel until the hospital consultation. For this reason, the number of patients captured in the most advanced stage of HY was very small, which limits the generalization of the results in this subgroup of patients. Another important limitation of our study is that it would be necessary to carry out an external validation in a different population to determine the predictive capacity. In this sense, it would be very interesting for other researchers to try to reproduce our study to know the extrapolation capacity of our predictive model.

However, as a strength, we highlight that this study is the first to develop a tool to predict quality of life based on relatively easy variables to obtain. Furthermore, the prediction results were excellent in the elaboration cohort, with an AUROC curve of >0.9. The predictive capacity at one year and two years, although slightly reduced, had AUROCs above 0.8.

## 5. Conclusions

Advanced HY stages, polypharmacy, being older, being a woman, being institutionalized, using a cane or a wheelchair, having more than five NMS, having a major neurocognitive disorder, and having a recognized disability percentage of >66% were correlated with a worse overall HRQoL in patients with PD. The predictive calculator will allow professionals to predict HRQoL in each PD patient and add supportive interventions to prevent or treat related factors.

## Figures and Tables

**Table 1:** Sociodemographic and clinical characteristics of the study sample.

Variable	T0 % (*n*)
Mean age (SD) 95% CI	69.51 (8.63) 68.14–70.88
Sex	
Male	59.4 (92)
Female	40.6 (63)
Living situation	
With family	85.8 (133)
Lives alone	9.7 (15)
Nursing home	4.5 (7)
Civil status	
Married	75.5 (117)
Widowed	16.1 (25)
Divorced	1.9 (3)
Employment situation	
Retired	73.5 (114)
Homemaker	19.4 (30)
Actively employed	5.2 (8)
Education	
No education	0.6 (1)
Primary level	82.6 (128)
Secondary level	12.3 (19)
University level	4.5 (7)
Disease duration	
≤ 5 years	29% (45)
6 to 10 years	36.1% (56)
11 to 15 years	18.8% (29)
16 years and older	15.8% (25)

SD, standard deviation, CI, confidence interval.

**Table 2:** Factors associated with quality of life in persons with Parkinson’s disease. Bivariate and multivariate analysis by linear regression.

Variable	Quality of Life Scores Mean (SD)	MD 95% CI *	aMD 95% CI **
Age (per one year)		**0.36 (0.07, 0.66)**	
Education level			
No education	50.26 (NC)	Ref.	
Primary level	28.89 (16.23)	−21.37 (−53.62, −10.88,)	
Secondary level	21.68 (13.58)	−28.58 (−57.85, 0.70,)	
University level	14.07 (10.74)	**−36.19 (−64.27, −8.11)**	
Sex			
Female	28.27 (14.82)	Ref.	Ref.
Male	26.93 (17.06)	−1.35 (−6.57, 3.88)	**−3.35 (−6.56, −0.46)**
Lives in nursing home			
No	26.60 (15.81)	Ref.	Ref.
Yes	45.91 (12.56)	**19.30 (7.31, 31.30)**	**9.05 (1.07, 17.04)**
Uses walking stick			
No	24.74 (15.52)	Ref.	Ref.
Yes	34.39 (15.79)	**9.66 (4.16, 15.14)**	**3.88 (0.09, 7.66)**
Uses crutches			
No	26.47 (16.02)	Ref.	
Yes	39.50 (12.91)	**13.03 (3.64, 22.42)**	
Uses a walker			
No	26.26 (16.12)	Ref.	
Yes	38.83 (11.65)	**12.58 (4.11, 21.04)**	
Uses a wheelchair			
No	26.17 (15.77)	Ref.	Ref.
Yes	44.54 (10.81)	**18.36 (8.79, 27.94)**	**7.46 (0.57, 14.34)**
Has a caregiver			
No	22.00 (13.22)	Ref.	
Yes	35.69 (16.73)	**13.70 (8.92, 18.47)**	
Years of Parkinson disease progress		0.91 (0.54, 1.28)	
HY Stage			
1–2	20.17 (11.97)	Ref.	Ref.
3–5	42.37 (13.06)	**22.20 (18.03, 26.37)**	**10.32 (5.82, 14.81)**
Surgery for neurostimulation			
No	26.31 (16.34)	Ref.	
Yes	31.99 (14.78)	5.68 (−0.61, 11.97)	
Has Alzheimer’s disease or major neurocognitive disorder			
No	26.21 (15.31)	Ref.	Ref.
Yes	51.53 (13.85)	**25.33 (13.66, 36.99)**	**14.16 (6.27, 22.05)**
More than two motor symptoms			
No	23.41 (16.00)	Ref.	
Yes	36.02 (12.89)	**12.62 (7.50, 17.73)**	
More than five non-motor symptoms			
No	19.61 (13.92)	Ref.	Ref.
Yes	35.45 (14.29)	**15.84 (11.36, 20.32)**	**8.26 (4.72, 11.81)**
Polypharmacy (>=4 medicines)			
No	23.11 (14.13)	Ref.	Ref.
Yes	33.69 (16.91)	**10.58 (5.64, 15.52)**	**6.38 (3.03, 9.74)**
Disability >66%			
No	23.35 (14.62)	Ref.	Ref.
Yes	39.75 (14.28)	**16.39 (11.07, 21.70)**	**7.50 (3.35, 11.65)**

MD: Mean difference; aMD: Adjusted mean difference by multivariate analysis; * Linear regression; ** Multiple linear regression. Statistically significant associations are indicated in bold.

## Data Availability

The data sets generated and/or analyzed during the current study are available from the corresponding author on reasonable request.
